# Influence of Temperature on the Natural Vibration Characteristics of Simply Supported Reinforced Concrete Beam

**DOI:** 10.3390/s21124242

**Published:** 2021-06-21

**Authors:** Yanxia Cai, Kai Zhang, Zhoujing Ye, Chang Liu, Kaiji Lu, Linbing Wang

**Affiliations:** 1 Beijing Zhonglu Gaoke Highway Technology Co., Ltd., Beijing 100088, China; 13311530926@163.com (Y.C.); kj.lu@rioh.cn (K.L.); 2 Research and Development Center of Transport Industry of New Materials, Technologies Application for Highway Construction and Maintenance, Beijing 100088, China; 3 Research Institute of Highway Ministry of Transport, Beijing 100088, China; 4 China Construction Third Bureau First Engineering Co., Ltd., Wuhan 430040, China; ygsbgs@cscec.com; 5 National Center for Materials Service Safety, University of Science and Technology Beijing, Beijing 100083, China; yezhoujing@ustb.edu.cn (Z.Y.); g20199178@xs.ustb.edu.cn (C.L.); 6 Virginia Tech, Blacksburg, VA 24061, USA

**Keywords:** temperature, natural vibration characteristics, simply supported beam, beam bridge, Midas/Civil

## Abstract

Natural vibration characteristics serve as one of the crucial references for bridge monitoring. However, temperature-induced changes in the natural vibration characteristics of bridge structures may exceed the impact of structural damage, thus causing some interference in damage identification. This study analyzed the influence of temperature on the natural vibration characteristics of simply supported beams, which is the most widely used bridge structure. The theoretical formula for the variation of the natural frequency of simply supported beams with temperature was proposed. The elastic modulus of simply supported beams in the range of −40 °C to 60 °C was acquired by means of the falling ball test and the theoretical formula and was compared with the elastic modulus obtained by the three-point bending test at room temperature (20 °C). In addition, the Midas/Civil finite-element simulation was carried out for the natural frequency of simply supported beams at different temperatures. The results showed that temperature was the main factor causing the variation of the natural frequency of simply supported beams. The linear negative correlation between the natural frequency of simply supported beams and their temperature were observed. The natural frequency of simply supported beams decreased by 0.148% for every 1 °C increase. This research contributed to the further understanding of the natural vibration characteristics of simply supported beams under the influence of temperature so as to provide references for natural frequency monitoring and damage identification of beam bridges.

## 1. Introduction

The natural vibration characteristics of bridges, including frequency, vibration mode, and damping, are affected by structural stiffness and the extent of damage, which can provide a reference basis for bridge design and comprehensive performance evaluation [1,2,3,4]. However, environmental factors, such as temperature, humidity, wind speed, and extremely harsh environments, can also cause significant changes in the natural vibration characteristics of the structure, which may even be greater than the changes in the natural vibration characteristics caused by actual damage, leading to difficulties in damage identification technologies for bridge monitoring [5,6,7,8,9].

As an important reference basis in damage identification, natural vibration characteristics play an essential role in bridge monitoring. In recent years, many researchers have explored the evolutionary law of natural bridge frequency and environmental factors. Farrar CR et al. [10] carried out a study on the modal parameters of beam bridges based on environmental factors and found that the natural frequency of beam bridges varied by 5% over 24 h. Therefore, it can be concluded that the bridge modal change is mainly related to the bridge temperature. Additionally, Liu and DeWolf [11] conducted a one-year observation on a curved concrete box girder bridge to investigate the dynamic characteristics. The results showed that temperature change had an impact on the bridge’s modal frequency, with a maximum of a 6% change in the bridge’s modal frequency when the temperature changed to 39 °C over a year. Through regular monitoring of the temperature and modal frequency of the reinforced concrete beam model, Liu et al. [5] found that, when the temperature experienced a change from −14.1 °C to 22.8 °C, the first four modal frequencies changed by 5–12%. This variation is sufficient to cover the variation in modal frequency due to structural damage. Sun Limin et al. [12] conducted research on the relationship between temperature, humidity, and bridge modal frequency based on the concrete continuous beam bridge model in the laboratory. The experimental results suggested that the natural frequency of the bridge decreased linearly with the increase of temperature. When the boundary condition remained constant, the influence of air humidity on the structure frequency was less than that of temperature. In addition, changes in temperature may alter the boundary conditions of the bridge and thus affect the structural frequency. The above studies proved that the temperature has a significant influence on the bridge’s natural frequency among the environmental factors. Therefore, it is necessary to investigate the effect of temperature on the natural vibration characteristics of bridges.

In order to study the influence of temperature on the natural vibration characteristics of bridges, the use of field monitoring of the bridge, or the finite-element method, is the main strategy to obtain the relationship between bridge natural frequency and temperature. However, the influence of other factors cannot be excluded in field monitoring of the bridge, resulting in different conclusions. Most of the studies [5,12,13,14,15,16,17,18] indicate that the frequency decreases with the increase of temperature, though there are also papers [19,20,21] that hold the opposite views. It should be noted that the long-term monitoring data cannot exclude the influence of material property changes and structural damage on natural vibration characteristics. Changes in the natural vibration characteristics of bridges are not necessarily caused by temperature. Changes in the self-vibration characteristics of bridges are also not necessarily caused by temperature, as there are many environmental factors simultaneously in effect. Furthermore, the influence of temperature on the dynamic characteristics of the bridge is different for various bridge types and boundary conditions, which is the main reason why some scholars reached different conclusions.

The reinforced concrete simply supported beam bridge is the earliest and most widely used bridge structure, which consists of beams supported by movable bearings and articulated bearings at both ends (namely: simply supported beam) as the main load-bearing structure. The factors that influence the natural frequency of simply supported beam bridges due to temperature changes are as follows [22,23]: (1) The change of temperature will cause the change in elastic modulus of structural materials (such as concrete and the reinforcing bar); (2) The change of temperature will cause changes in structure size; (3) In the statically indeterminate structure, the change of temperature can cause secondary stress; and (4) The change of temperature will cause changes in boundary conditions of the structure, which are mainly manifested by changes in abutment stiffness. Yang [24] and Li [25] both analyzed the influence of ambient temperature on the natural vibration characteristics of concrete beams through laboratory experiments, and proposed that there is a negative correlation between the bridge’s natural frequency and the ambient temperature. However, both experiments were performed at ambient temperature, which, in addition to the temperature of the simply supported beam have relatively small changes with the temperature control range above 0 °C. It is necessary to conduct experiments over a wider range of temperature variations so as to ensure the universality and reliability of the conclusions.

In this paper, the influence of temperature on the natural vibration characteristics of reinforced concrete simply supported beam was analyzed in the laboratory. The theoretical calculation formula for simply supported beam natural frequency variation with temperature was presented. The variation law of the natural frequency of testing beams within the temperature range of −40 °C to 60 °C was explored by means of the falling ball test and three-point bending test. The Midas/Civil finite-element simulation method was also applied to analyze the natural frequency of the testing beam at different temperatures. Through theoretical analysis, experimental analysis, and simulation analysis, the relationship between the natural frequency and the temperature was derived, which can provide references for actual bridge natural frequency monitoring and damage identification.

## 2. Theoretical Analysis on the Influence of Temperature on the Natural Frequency of Simply Supported Beam

As the simply supported beam is a statically determinate structure without abutment stiffness, temperature will not cause changes in secondary structural stress and boundary conditions. The influence of temperature on elastic modulus and structure size is analyzed below.

### 2.1. Influence of Temperature on Elastic Modulus

The influence of temperature on a concrete elastic modulus far exceeds that of a reinforced elastic modulus. Concrete is the main material of the simply supported beam. Therefore, the influence of temperature on reinforcing bar was ignored. The analysis of the influence of temperature on the elastic modulus of the simply supported beam was in accordance with the calculation formula in the European Concrete Specification CEB-FIP Model Code 2010 [26]:

(1) $$E_{T}=E_{20^{{}^{\circ}}C}\left[1-\theta_{E}\left(T-20\right)\right]$$

where,

$E_{T}$: Elastic modulus (Mpa) of concrete at T temperature;
T: Temperature of concrete (°C);
$E_{20^{{}^{\circ}}C}$: Elastic modulus of concrete (MPa) at 20 °C;
$\theta_{E}$: Temperature coefficient of elastic modulus, with a value of 0.003.

### 2.2. Influence of Temperature on Structure Size

The influence of temperature on the structure size of the simply supported beam is mainly manifested in the elongation or shortening of the beam and the change of the section inertia moment, which is related to the properties of the material. For the rectangular equal section simply supported beam, the change in beam length can be obtained by the following formula:

(2) $$l_{l}=\left(1+\alpha \Delta T\right)l$$

where,

$l_{1}$: Beam length after temperature change;
α: Linear expansion coefficient with reinforcement of 1.2 × 10^−6^ and concrete of 1.0 × 10^−5^;
ΔT: Temperature change;
l: Initial beam length;

The variation of beam width b and beam height h can be expressed as:

(3) $$b_{l}=\left(1+v\alpha \Delta T\right)b$$

(4) $$h_{l}=\left(1+v\alpha \Delta T\right)h$$

where, v represents Poisson’s ratio, with concrete Poisson’s ratio v=0.2 and reinforced Poisson’s ratio v=0.31. On the basis of section inertia moment $I=\frac{{\mathrm{bh}}^{3}}{12}$, the variation of the section inertia moment can be expressed as:

(5) $$I=\frac{{\left(1+v\alpha \Delta T\right)}^{4}{\mathrm{bh}}^{3}}{12}$$

### 2.3. Influence of Temperature on the Natural Frequency of Simply Supported Beam

**Figure 1:**
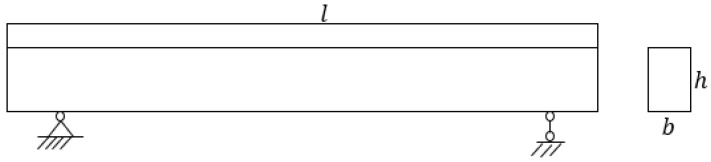
Diagram of the simply supported beam.

According to the derivation method in the literature [2,17,23], the relationship between the natural frequency and the temperature was explored using the first-grade rectangular section simply supported beam as a model. Let the length, width, and height be l, b, and h respectively, as shown in Figure 1.

The basic transverse vibration equation for the undamped homogeneous beam is as follows [2,17]:

(6) $$\frac{\partial^{4}v\left(x,t\right)}{\partial x^{4}}+\frac{\rho A}{\mathrm{EI}}\times \frac{\partial^{2}v\left(x,t\right)}{\partial t^{2}}=0$$

The formula for the free vibration frequency of the model is:

(7) $$f_{n}=\frac{n^{2}\pi }{2l^{2}}\sqrt{\frac{\mathrm{EI}}{\rho A}}$$

where,

$f_{n}$: nth order modal frequency
l: Calculated span lengths of beam
E: Young’s modulus
I: Second moment of area
ρ: Mass density
A: Sectional area

Standard vibration mode function:

(8) $$\varphi_{n}\left(x\right)=\sin\frac{n\pi x}{l}$$

The fundamental frequency temperature is 20 °C. Function (1) is adopted, considering the change of the material elastic modulus with temperature:

(9) $$E_{T}=E\left(1-\theta_{E}\Delta T\right)$$

By substituting Formulas (2), (5), and (9) into Formula (7), the natural frequency of the simply supported beam considering the effect of temperature is:

(10) $$f_{n}^{T}=\frac{n^{2}\pi }{2l^{2}}\sqrt{\frac{\mathrm{EI}}{\rho A}}\cdot \frac{\sqrt{\left(1-\theta_{E}\Delta T\right)}{\left(1+v\alpha \Delta T\right)}^{2}}{{\left(1+\alpha \Delta T\right)}^{2}}=\lambda_{I}\lambda_{l}\lambda_{E}\frac{n^{2}\pi }{2l^{2}}\sqrt{\frac{\mathrm{EI}}{\rho A}}$$

where,

$\lambda_{I}$: Influence coefficient of inertia moment, $\lambda_{I}={\left(1+v\alpha \Delta T\right)}^{2}$
$\lambda_{l}$: Influence coefficient of beam length, $\lambda_{l}=\frac{1}{{\left(1+\alpha \Delta T\right)}^{2}}$
$\lambda_{E}$: Influence coefficient of elastic modulus, $\lambda_{E}=\sqrt{1-\theta_{E}\Delta T}$

With model beam $\alpha =1.0\times 10^{-5}$, v =0.2, and $\theta_{E}=0.003$ into Function (10), the change rate of the natural frequency of the simply supported reinforced concrete beam is:

(11) $${\Delta f}_{n}=\left(\frac{f_{n}^{T}}{f_{n}}-1\right)\times 100\%=\frac{\sqrt{\left(1-0.003\Delta T\right)}{\left(1+0.2\times 10^{-5}\Delta T\right)}^{2}}{{\left(1+1.0\times 10^{-5}\Delta T\right)}^{2}}-1$$

**Figure 2:**
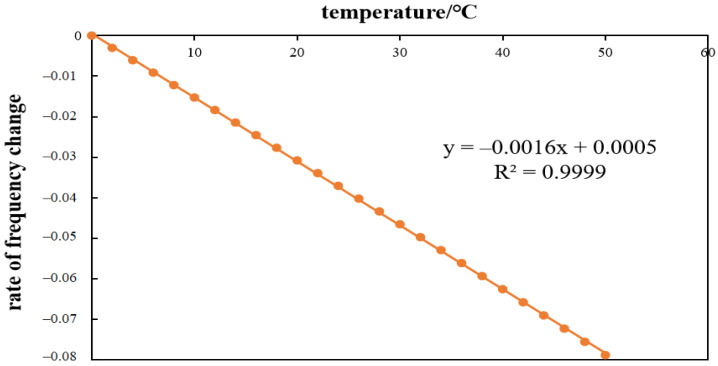
The relationship between $\Delta f_{n}$ and ΔT.

Taking 0–50 °C with the interval of 2 °C and assigning the value to ΔT in Formula (11), the corresponding value of $\Delta f_{n}$ can be obtained. The curve fitting is shown in Figure 2.

It can be seen from Figure 2 that R^2^ = 0.9999, which indicates that the fitting curve error was small, and $\Delta f_{n}=-0.16\%$, which means that the frequency change of the model beam caused by the change of material characteristics and geometric size was −0.16% when the temperature rose by 1 °C. Taking 20 °C as the fundamental frequency temperature, the influence coefficients of each grade of natural frequency in the ultimate temperature drop of 60 °C were obtained as follows:

Influence coefficient of elastic modulus: $\lambda_{E}$ = 1.08628
Influence coefficient of inertia moment: $\lambda_{I}$ = 0.99976
Influence coefficient of beam length: $\lambda_{l}$ = 1.00120

Considering the ultimate cooling ΔT=60 °C, the elastic modulus of the simply supported beam causes the frequency to increase by 8.628%; the section inertia moment causes the frequency to decrease by 0.024%; the beam length causes the frequency to increase by 0.12%; and the comprehensive effect of structure size causes the frequency to increase by 0.096%. In comparison, the changes in section inertia moment and beam length are negligible. The change in the natural frequency of the simply supported beam with temperature is mainly related to the material elastic modulus, while the change in the structure size has almost no effect on the frequency.

## 3. Experimental Study on the Influence of Temperature on the Natural Frequency of Simply Supported Beam

### 3.1. Test Scheme

**Figure 3:**
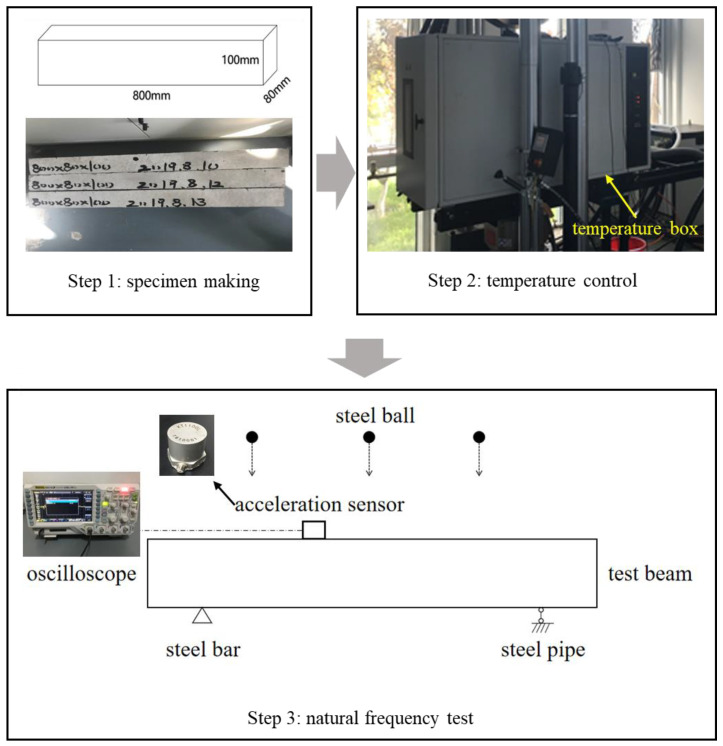
Test scheme.

The test scheme is shown in Figure 3, and was mainly divided into three steps.

Step 1: Three reinforced concrete testing beams made of C30 concrete and HRB400 reinforcing bar were prefabricated, with lengths of 800 mm, widths of 80 mm, and heights of 100 mm.

Step 2: The temperature of testing beams was controlled. The No. 1 and No. 2 testing beams were placed into the temperature box as parallel test models, and the No. 3 testing beam was reserved. The model of the temperature box was water+bai ag ETC 550-1, with the working temperature of −150 °C to 600 °C. The temperature in the temperature box was adjusted to 20 °C. To ensure that the internal temperature of the testing beam was consistent with the external temperature, it was kept at a constant temperature for 3 h after the temperature box reached 20 °C. In order to make the test results more accurate, the test was carried out immediately after the No. 1 testing beam was taken out, while the No. 2 testing beam was insulated in the temperature box.

Step 3: First, the testing beam was fixed. At 100 mm from the left and right ends of the testing beam, steel bars with a diameter of 20 mm were respectively used as a fixed end abutment and a sliding end abutment. Before the testing, the upper end of the testing beam was divided into four equal lengths. The center position of the three bisectors was marked as the landing point of small balls. Then, the acceleration sensor (Type: KT1100L, Measurement range:5 g) was fixed with silicone rubber at a distance of 300 mm from one end of the testing beam. The oscilloscope (Type: RIGOL MSO1104) was adjusted to the “SINGLE” state. By controlling the air compressors (Type: AFS-550), the ball was fixed by the fixture. The ball fell freely onto the testing beam. After landing, the height was adjusted until the oscilloscope showed a smooth fluctuation curve. By controlling the air compressors, the ball was dropped onto the testing beam from three landing points in turn to obtain three waveforms. Then, the same test was carried out on the No. 2 testing beam. Finally, the waveform diagrams of the No. 1 and No. 2 testing beams at −40 °C, −20 °C, 0 °C, 20 °C, 40 °C, and 60 °C were sequentially measured.

### 3.2. Result Analysis

**Figure 4:**
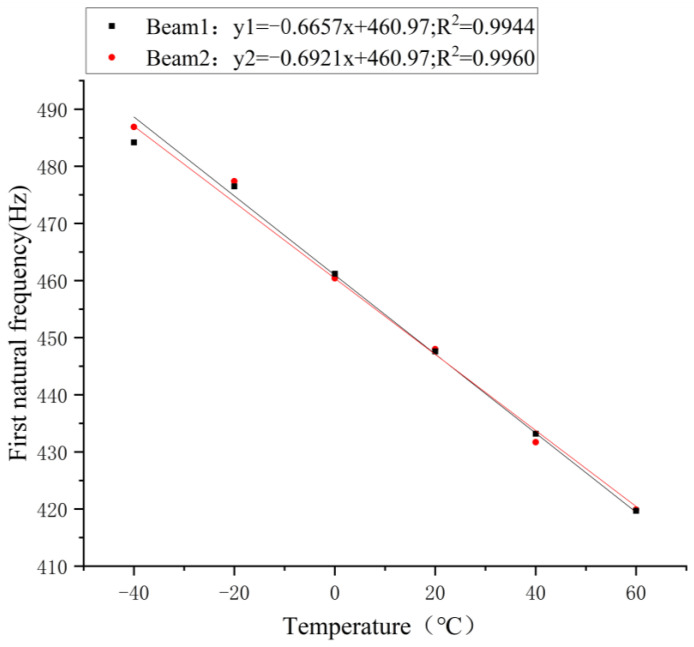
Relationship between the first-grade natural frequency of the No. 1 beam and the No. 2 beam with temperature change.

According to the average value of the natural frequency obtained after three drops of balls fall at the same temperature, the experimental results of the natural frequency at different temperatures were obtained, as shown in Figure 4.

Figure 4 shows the temperature-frequency fitting curve. R^2^ of the No. 1 beam and No. 2 beam were both close to 1, indicating a good fit. Essentially, there was a linearly negative correlation between the natural frequency of the testing beam and the temperature. In addition, the results of the No. 1 and No. 2 testing beams were essentially the same. When the temperature increased by 1 °C, the fundamental frequency of the No. 1 testing beam decreased by 0.6657 Hz, with the change rate of −0.149%. When the temperature increased by 1 °C, the fundamental frequency of the No. 2 testing beam decreased by 0.6921 Hz, with the change rate of −0.154%. The above results are similar to the natural frequency change rate of −0.16% obtained by the theoretical formula, indicating that the experimental results were consistent with the theoretical formula.

**Figure 5:**
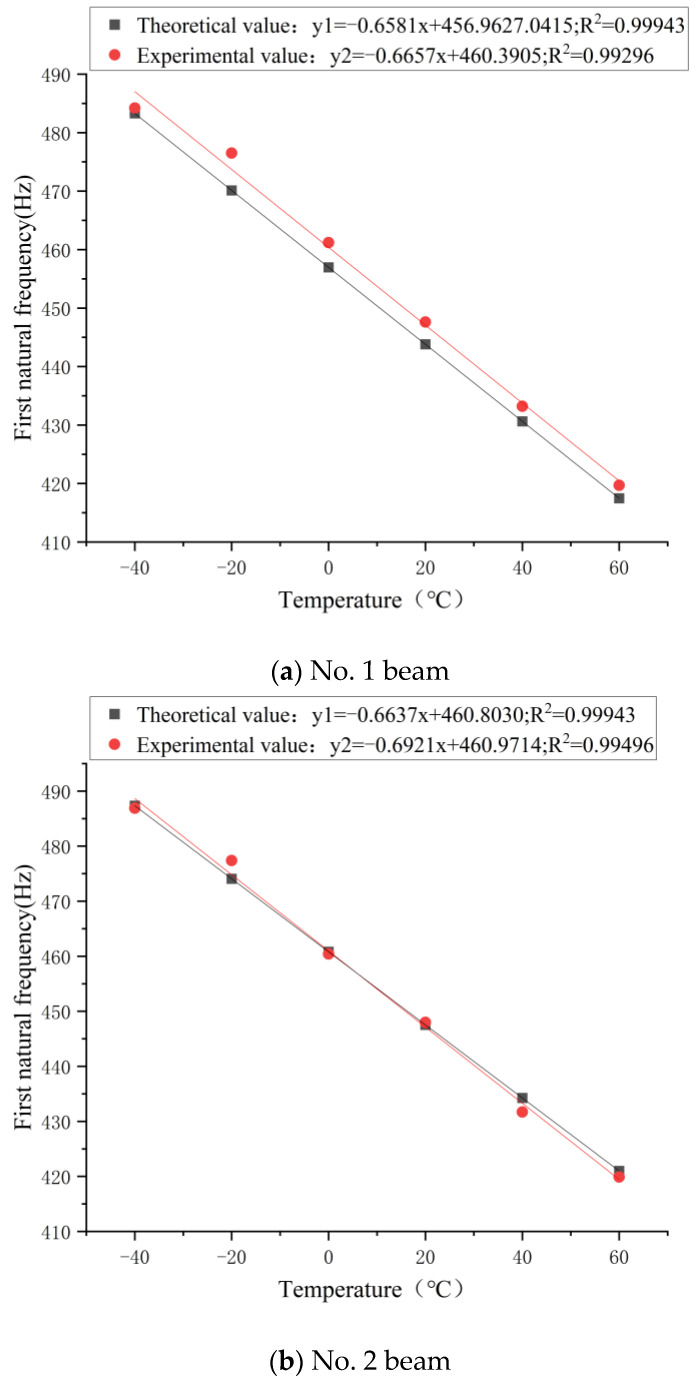
Relationship between theoretical results of the testing beam and experimental results of the first-grade natural frequency with temperature change.

Figure 5 shows the comparison between the theoretical and experimental results of the first-grade natural frequency of the No. 1 and No. 2 testing beams at different temperatures. Among them, the theoretical calculation results are derived from Formula (10).

In Figure 5, there is little difference in the slopes between the experimental value and the theoretical value of the fitting curve of the No. 1 and No. 2 beams, which means that the change rate of the first-grade frequency with temperature is essentially the same. Therefore, it can be concluded that the experimental results are consistent with the theoretical formula. The test value of the No. 1 beam at −40 °C had a large deviation from the fitted curve, which may have been caused by the temperature increase of the test beam when the test beam was taken out from the −40 °C environment into the normal temperature environment and because the experiment was not carried out immediately. Additionally, the non-linearity of natural frequency below 0 °C increased with the decreasing temperature, resulting in a decrease in the linear dependence of the data points overall. This may have been due to the moisture of the concrete beam. The moisture in concrete undergoes a phase change at around 0 °C, which may have an impact on the natural frequency of concrete. The influence of water should not be omitted when the concrete is subjected to lower temperatures. When the temperature was between 20 °C and 60 °C, the error between the theoretical value and the experimental value may have been caused by the inevitable error in the experimental process and the insufficient consideration of factors in the theoretical formula. Figure 5 illustrates that the theoretical formula of the temperature influence coefficient is applicable to the calculation of the first-grade frequency of the simply supported beam.

### 3.3. Method Verification

The elastic modulus of the testing beam can be derived from the theoretical Formula (7) based on frequency and temperature. Therefore, the accuracy of the frequency-temperature test results can be verified by comparing the theoretical value with the elastic modulus of the testing beam measured by the three-point bending test.

**Figure 6:**
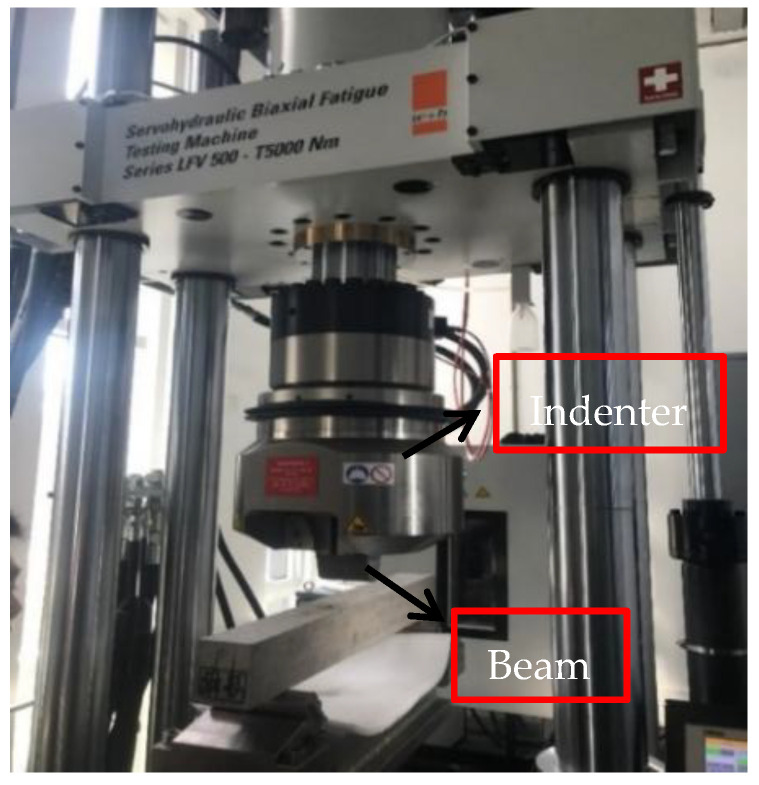
Three-point bending test.

In this paper, the three-point bending test was used to determine the elastic modulus. The fatigue testing machine was used to carry out the concentrated loading and mass values of the No.1 and No.2 testing beams within the elastic range at a room temperature of 20 °C. The test results are shown in Figure 6.

The elastic modulus of the testing beam can be obtained by the following formula:

(12) $$E=\frac{F}{\Delta L}\cdot \frac{l^{3}}{48I}$$

where,

F: Concentrated loading (N);
ΔL: Deflection (mm);
l: Testing beam effective span length (m);
I: Section inertia moment (m^4^);

According to Formula (12), the test results are shown in Table 1 and Table 2.

The elastic modulus calculated from the deflection value is the actual elastic modulus in the three-point bending test. The error between the calculated value and the actual value in the frequency-temperature test of the elastic modulus of the testing beam was relatively small, indicating that the results of frequency-temperature test were correct.

## 4. Simulation Analysis on the Influence of Temperature on the Natural Frequency of Simply Supported Beam

### 4.1. Model Establishment

**Figure 7:**
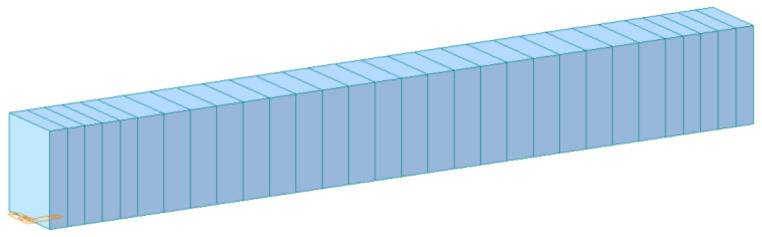
The testing beam model.

Midas/Civil is a special finite-element software for general structural analysis and design systems in the bridge field, which is mainly applied to the linear-elastic small deformation beam element calculations. Midas/Civil was used to create the testing beam model, with size and structure consistent with the testing beam. The *Y* axis is the width direction, the *X* axis is the length direction, and the *Z* axis is the height direction. The calculated span length of the beam refers to the distance between two supports [27,28,29]. Considering the distance between supports is 600 mm, the calculated span length of the beam during the numerical simulation was set to 600 mm. The element type of the finite-element model is the beam element, in which a beam element can consist of two nodes. The model had a total of 30 beam elements with simply supported beam boundary conditions, which is shown in Figure 7.

In order to make the simulation results more accurate, the actual values of the elastic modulus at 20 °C obtained from the three-point bending test were substituted into the design parameters of the testing beam model and verified with Formula (1).

### 4.2. Result Analysis

**Figure 8:**
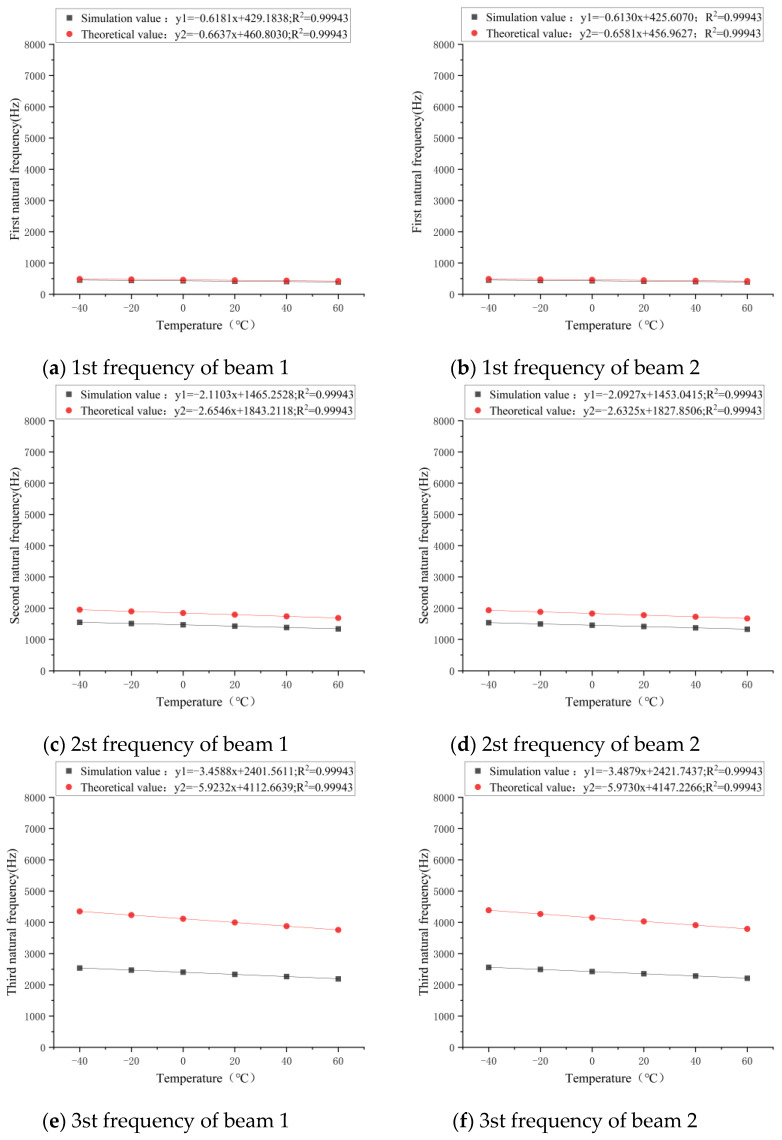

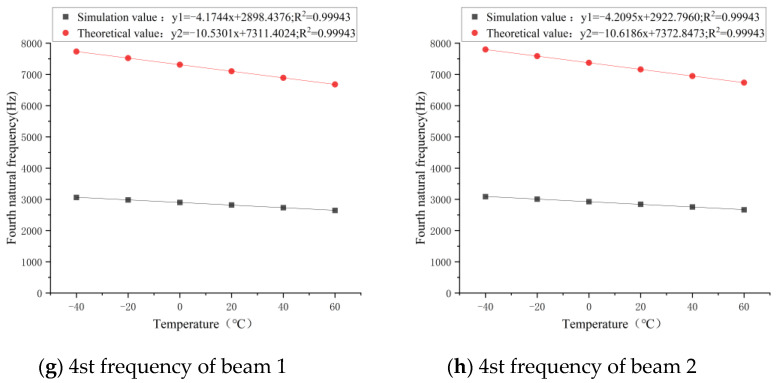
Relationship between the n-grades natural frequency (n = 1, 2, 3, 4) and temperature of the testing beam’s theoretical and simulation results.

The first four-grades natural frequency of the testing beam model obtained at a fundamental frequency temperature of 20 °C were taken. Then, the first four-grades natural frequency of the testing beam model at −40 °C, −20 °C, 0 °C, 20 °C, 40 °C, and 60 °C was successively obtained. The relationship between n-grades natural frequency (n = 1, 2, 3, 4) and temperature in the theoretical results and simulation results of beam 1 and beam 2 is shown in Figure 8.

According to Figure 8a,b, the residual square and R^2^ of temperature-frequency fitting curve of the No. 1 beam and No. 2 beam were both close to one, indicating that fitting was good. Both the theoretical and simulation results showed that the first four-grades modal frequency of the testing beam were linearly negatively correlated with temperature. According to the frequency-temperature fitting curve, the theoretical and simulated values of the first-grade natural frequency of the No. 1 beam and No. 2 beam had little difference within the temperature range of −40 °C to 60 °C, which were basically consistent. In terms of the change rate, when the temperature of the No.1 beam increased by 1 °C, the theoretical value decreased by 0.148%, and the simulated value decreased by 0.149%. The results were essentially the same for both. When the temperature of the No. 2 beam increased by 1 °C, the theoretical value decreased by 0.148%, and the simulated value decreased by 0.150%. The results of both are also almost the same.

Figure 8c,d shows the relationship between the theoretical and simulated second-order natural frequency and temperature of the No. 1 beam and No. 2 beam, respectively. According to the frequency-temperature fitting curve, it was found that the theoretical value of the second-order natural frequency was greater than the simulated value in the temperature range of −40~60 °C. At 20 °C, the theoretical value of the No. 1 beam was 1777.306 Hz, and the simulated value was 1412.862 Hz. Under the same temperature conditions, the theoretical value of the No.2 beam was 1792.243 Hz, and the simulated value was 1424.735 Hz. Based on the theoretical value, the simulated value was about 20% smaller than the theoretical value, indicating that there was a relatively large error. In terms of the change rate, for every 1 °C increase in temperature, the theoretical value decreased by 0.148% and the simulated value decreased by 0.148%. That is to say, the two were basically consistent in terms of the change trend.

Figure 8e–h show the relationship between theoretical and simulated third-order and fourth-order natural frequencies and temperature of the No. 1 beam and No. 2 beam. From the frequency-temperature fitting curve, it was found that the variation trend of the third-order natural frequency and fourth-order natural frequency of the No. 1 beam was similar to that of the second-order natural frequency in the temperature range of −40~60 °C. In addition, the slope of the curve increased with the increase of order. However, the theoretical increase in the slope of the curve was much greater than the simulated one. At 20 °C, the theoretical value of the third-order natural frequency of the No.1 beam was 3998.94 Hz, and the simulated value was 2335.15 Hz. In contrast, the simulated value was 41.61% lower than the theoretical value. At the same temperature, the theoretical value of the fourth-order natural frequency was 7109.23 Hz, and the simulated value was 2818.29 Hz. In contrast, the simulated value was 60.36% lower than the theoretical value. Furthermore, the comparison result of the No. 2 beam was also similar. With the theoretical value as the standard, the high-order natural frequency of the beam will cause a large error. According to the theoretical Formula (7), it was found that the n-order frequency of the simply supported beam was n^2^ times the first-order frequency. Although the first-order frequency obtained by theoretical calculation was close to the simulated first-order frequency, there were still errors. With the increase of the order, the error between the theoretical frequency and the simulated frequency will further increase. However, the simulation results had the same trend as the theoretical results. In other words, there was a linear relationship between frequency and temperature change. The simulation results showed that the theoretical calculation formula is only applicable for calculating the first-grade frequency of the simply supported beam, not for the frequencies above the first grade, which provides references for the study on natural vibration characteristics of actual bridges.

## 5. Conclusions

This paper obtained the relationship between the natural frequency of simply supported beams and temperature through theoretical analysis, experimental testing, and numerical simulation. The conclusions are as follows:

(1) The theoretical calculation formula for the change of natural frequency with temperature was obtained through the theoretical analysis of natural vibration characteristics of simply supported reinforced concrete beams. Theoretical analysis indicated that the effect of temperature on the natural frequency of the simply supported beam was mainly caused by the change of the elastic modulus of simply supported beam material. There was little influence of structure size on natural frequency.
(2) The influence of temperature in the range of −40~60 °C on the natural frequency of the simply supported beam was analyzed through the small ball excitation test. The temperature range was so wide that it covered the conventional service temperature range of simply supported beam bridges. An acceleration sensor was used to monitor the natural frequency of the simply supported beam. The natural frequency-temperature change curve was obtained and verified. The curve showed that, for each 1 °C increase in temperature in the range of −40 °C to 60 °C, the natural frequency of the simply supported beam decreased by 0.148%.
(3) The Midas/Civil finite-element simulation was applied to analyze the natural frequency of simply supported beams at different temperatures. By comparing with the theoretical values, the theoretical calculation formula for the natural frequency of the simply supported beam was only applicable to the fundamental frequency, rather than the frequencies above the first grade.

The evolution law of the natural frequency of reinforced concrete simply supported beams with temperature changes can provide references for dynamic characteristics monitoring and damage identification of simply supported beam bridges. The actual bridge structure and the influence of uneven temperature distribution on the bridge’s natural vibration characteristics need to be considered in subsequent studies.

## Figures and Tables

**Table 1:** Elastic modulus of testing beam.

Beam Number	Initial Deflection/ mm	Initial Load/ N	Deflection/ mm	Load/ N	Elastic Modulus/ 10^^4^ MPa
1	−0.0005	0	−0.0180	−803.36	3.0986
2	0	0	−0.0195	−910.26	3.1509

**Table 2:** Comparison of the elastic modulus measured by different methods (20 °C).

Beam Number	Elastic Modulus/10^^4^ MPa		
	Frequency-Temperature Test Calculation Value	Three Point Bending Test Value	Relative Error
1	3.1569	3.0986	1.88%
2	3.1626	3.1509	0.37%

## Data Availability

Data is contained within the article.
